# Supplementary material for: Macroecological patterns in experimental microbial communities
Source: PLoS Comput Biol. 2025 May 8;21(5):e1013044. doi: 10.1371/journal.pcbi.1013044 (PMC12112161; doi:10.1371/journal.pcbi.1013044)
Supplement: S1 Text — Additional information about the community assembly experiment (PDF) [file pcbi.1013044.s001.pdf]

Macroecological patterns in experimental microbial communities: S1 Text

William R. Shoemaker[1,*], Álvaro Sánchez[2], and Jacopo Grilli[1]

1 Quantitative Life Sciences, The Abdus Salam International Centre for Theoretical Physics (ICTP), Trieste, 34151, Italy.
2 Instituto de Biología Funcional y Genómica, IBFG-CSIC, Universidad de Salamanca, 37007, Salamanca, Spain.

*** Contact:** williamrshoemaker@gmail.com

S1 Text: Additional experimental details

Experimental data was obtained from a previous study where a large number of replicate ecological communities originating from a single progenitor soil sample were propagated in controlled laboratory conditions under regional or global migration treatments (Table S1; [1]). A given replicate community was initiated by inoculating 4 μL (low inoculum) or 40 μL (high inoculum) of the source community into 500 μL of M9 minimal media with 0.2% glucose into a well of a 96 deep-well plate (VWR) at 30°C under static conditions. Transfers were performed every 48 hours, 18 times in total for all replicate communities with a dilution rate $D_{\mathrm{transfer}} = 0.0084$ (Table 1).

The transfer procedure was modified to manipulate the effects of different forms of migration. For the regional migration treatment, a 4 μL aliquot of the soil supernatant was added to the 4 μL aliquot from the previous transfer. For the global migration treatment, 4 μL aliquots of all replicate populations were pooled, resuspended, and diluted 10,000-fold. A 4 μL aliquot of the diluted solution was added to the 4 μL aliquot from the previous transfer for each replicate community. At the end of each 48 h. transfer period samples from each replicate community were mixed with 40% glycerol, cryopreserved at -80°C, and DNA was extracted and sequenced.

DNA extraction was performed using a QIAGEN DNeasy 96 Blood and Tissue kit. Library preparation for 16S rRNA amplicon sequencing of the V4 region was performed as previously described [2] and PCR products were purified and normalized using the SequalPrep PCR kit (Invitrogen). Sequencing was performed on an Illumina MiSeq (2x250 bp paired-end) and raw reads were processed for demultiplexing and barcode, index, and primer removal using QIIME v1.9 [3]. The number of communities sequenced at each transfer for each treatment can be found in Table S1. In this study we reprocessed all raw FASTQ data from the original study to obtain Amplicon Sequence Variants (ASVs) using `DADA2` [4]. We reprocessed the data using the pooled option inference option so that ASVs with an abundance of one in a given community (i.e., singletons) could be inferred, allowing us to examine the entirety of the empirical sampling distribution. The attractor status of a given replicate community was assigned as previously described [1]. Additional detail can be found in the study that originally presented the experiment [1].

References

1. Sylvie Estrela, Jean C. C. Vila, Nanxi Lu, Djordje Bajić, Maria Rebolleda-Gómez, Chang-Yu Chang, Joshua E. Goldford, Alicia Sanchez-Gorostiaga, and Alvaro Sanchez. Functional attractors in microbial community assembly. *Cell Systems*, 13(1):29–42.e7, January 2022.

2. Joshua E. Goldford, Nanxi Lu, Djordje Bajić, Sylvie Estrela, Mikhail Tikhonov, Alicia Sanchez-Gorostiaga, Daniel Segrè, Pankaj Mehta, and Alvaro Sanchez. Emergent simplicity in microbial community assembly. *Science*, August 2018. Publisher: American Association for the Advancement of Science.

3. J. Gregory Caporaso, Justin Kuczynski, Jesse Stombaugh, Kyle Bittinger, Frederic D. Bushman, Elizabeth K. Costello, Noah Fierer, Antonio Gonzalez Pena, Julia K. Goodrich, Jeffrey I. Gordon, Gavin A. Huttley, Scott T. Kelley, Dan Knights, Jeremy E. Koenig, Ruth E. Ley, Catherine A. Lozupone, Daniel McDonald, Brian D. Muegge, Meg Pirrung, Jens Reeder, Joel R. Sevinsky, Peter J. Turnbaugh, William A. Walters, Jeremy Widmann, Tanya Yatsunenko, Jesse Zaneveld, and Rob Knight. QIIME allows analysis of high-throughput community sequencing data. *Nature Methods*, 7(5):335–336, May 2010. Bandiera_abtest: a Cg_type: Nature Research Journals Number: 5 Primary_atype: Correspondence Publisher: Nature Publishing Group Subject_term: Microbial ecology;Next-generation sequencing;Software Subject_term_id: microbial-ecology;next-generation-sequencing;software.

4. Benjamin J. Callahan, Paul J. McMurdie, Michael J. Rosen, Andrew W. Han, Amy Jo A. Johnson, and Susan P. Holmes. DADA2: High-resolution sample inference from Illumina amplicon data. *Nature Methods*, 13(7):581–583, July 2016. Bandiera_abtest: a Cg_type: Nature Research Journals Number: 7 Primary_atype: Research Publisher: Nature Publishing Group Subject_term: Metagenomics;Software;Statistical methods Subject_term_id: metagenomics;software;statistical-methods.
