# Supplementary material for: Macroecological patterns in experimental microbial communities
Source: PLoS Comput Biol. 2025 May 8;21(5):e1013044. doi: 10.1371/journal.pcbi.1013044 (PMC12112161; doi:10.1371/journal.pcbi.1013044)
Supplement: S2 Text — Definition of relative error. (PDF) [file pcbi.1013044.s002.pdf]

Macroecological patterns in experimental microbial communities: S2 Text

William R. Shoemaker[1,*], Álvaro Sánchez[2], and Jacopo Grilli[1]

1 Quantitative Life Sciences, The Abdus Salam International Centre for Theoretical Physics (ICTP), Trieste, 34151, Italy.
2 Instituto de Biología Funcional y Genómica, IBFG-CSIC, Universidad de Salamanca, 37007, Salamanca, Spain.

* **Contact:** `williamrshoemaker@gmail.com`

S2 Text: Error estimates

Error between observed and predicted quantities was estimated as the relative error.

$$\varepsilon = \left| \frac{\text{Obs.} - \text{Pred.}}{\text{Obs.}} \right| \tag{A}$$

The difference in relative error between two treatments was estimated as

$$\overline{\Delta \varepsilon} = \frac{1}{S_{\text{obs}}} \sum_{i=1}^{S_{\text{obs}}} \log \left[\frac{\varepsilon_{\text{mig.}}}{\varepsilon_{\text{no mig.}}} \right] \tag{B}$$
