# Supplementary material for: Macroecological patterns in experimental microbial communities
Source: PLoS Comput Biol. 2025 May 8;21(5):e1013044. doi: 10.1371/journal.pcbi.1013044 (PMC12112161; doi:10.1371/journal.pcbi.1013044)
Supplement: S3 Text — Derivation of logistic growth in a batch culture experimental setup. (PDF) [file pcbi.1013044.s003.pdf]

Macroecological patterns in experimental microbial communities: S3 Text

William R. Shoemaker[1,*], Álvaro Sánchez[2], and Jacopo Grilli[1]

1 Quantitative Life Sciences, The Abdus Salam International Centre for Theoretical Physics (ICTP), Trieste, 34151, Italy.
2 Instituto de Biología Funcional y Genómica, IBFG-CSIC, Universidad de Salamanca, 37007, Salamanca, Spain.

*** Contact:** `williamrshoemaker@gmail.com`

S3 Text: Phenomenological logistic growth from a consumer-resource model

We provide a heuristic explanation for how a deterministic phenomenological model of logistic growth can serve as a useful model for a population in a closed system with an initial concentration of supplied resources (i.e., a given transfer cycle illustrated by Fig 3). We start with a system of equations where one community member of abundance x consumes a single resource c to grow at rate $r(c)$

$$\frac{dx}{dt} = r(c)x \tag{Aa}$$

$$\frac{dc}{dt} = -\frac{r(c)x}{Y} \tag{Ab}$$

where Y represents the cell yield per-unit resource and the growth rate can be represented as Monod kinetics $r(c) = r_{\max}\frac{c}{c+K_c}$ where $r_{\max}$ is the maximum possible rate of growth and K_c is the half-saturation constant where $r(c)/r_{\max} = \frac{1}{2}$. We can obtain a single equation of logistic growth by 1) using the principle of mass conservation and 2) assuming that the half-saturation constant is sufficiently large relative to the initial concentration of supplied resources ($K_c \gg c(0)$). Under the principle of mass conservation, resources and yield-corrected abundances must sum to a constant total mass at any given time $B \equiv \frac{x(t)}{Y} + c(t)$. This constraint allows us to obtain a function for $c(t)$. The assumption $K_c \gg c(0)$ linearizes the growth rate $r(c) \approx r_{\max}c/K_c$. Using these two results, we obtain the following single differential equation

$$\frac{dx}{dt} = \frac{Br_{\max}}{K_c}x\left(1 - \frac{x}{YB}\right) \tag{Ba}$$

$$= \frac{x}{\tilde{\tau}}\left(1 - \frac{x}{\tilde{K}}\right) \tag{Bb}$$

where we have defined the timescale of growth and the carrying capacity, the terms of logistic growth, in mechanistic terms $\tilde{\tau} \equiv \frac{K_c}{Br_{\max}}$ and $\tilde{K} \equiv YB$. Therefore, a model of logistic growth is appropriate for microbial microcosm experiments provided that the half-saturation constant is sufficiently large relative to the supplied concentration of resources, a requirement that can, in principle, be manipulated by the experimenter.
