# Supplementary material for: Macroecological patterns in experimental microbial communities
Source: PLoS Comput Biol. 2025 May 8;21(5):e1013044. doi: 10.1371/journal.pcbi.1013044 (PMC12112161; doi:10.1371/journal.pcbi.1013044)
Supplement: S4 Text — Derivation of the stationary distribution of abundance for the SLM with a constant rate of migration. (PDF) [file pcbi.1013044.s004.pdf]

Macroecological patterns in experimental microbial communities: S4 Text

William R. Shoemaker[1,*], Álvaro Sánchez[2], and Jacopo Grilli[1]

1 Quantitative Life Sciences, The Abdus Salam International Centre for Theoretical Physics (ICTP), Trieste, 34151, Italy.
2 Instituto de Biología Funcional y Genómica, IBFG-CSIC, Universidad de Salamanca, 37007, Salamanca, Spain.

*** Contact:** `williamrshoemaker@gmail.com`

S4 Text: Deriving the stationary AFD for the SLM with a constant rate of migration

To determine the extent that continuous experimental manipulations of demography can alter the macroecological patterns captured by Eq. 1, we incorporate a migration term (m_i). By performing a change of variables for the relative abundance on the Langevin governing the dynamics of absolute abundance, we obtain

$$\frac{dx_i}{dt} = m_i + \frac{x_i}{\tau_i}\left(1 - \frac{x_i}{K_i}\right) + \sqrt{\frac{\sigma_i}{\tau_i}} x_i \xi_i(t) \tag{A}$$

Where m_i has dimensionless units and represents the number of individuals immigrating divided by the total number of individuals in the focal community, $m_i \equiv m_i^{(n)}/N$. It's clear that $\langle x_i \rangle \to K_i + m_i$ as $t \to \infty$. As in the main manuscript, we will use the Itô $\leftrightarrow$ Fokker-Planck correspondence to formulate a partial differential equation for the probability $P(x_i, t)$ that the ith species has abundance x at time t [1]

$$\frac{\partial P}{\partial t} = -\frac{\partial}{\partial x_i}\left[\left(m_i + \frac{x_i}{\tau_i}\left(1 - \frac{x_i}{K_i}\right)\right) P(x_i, t)\right] + \frac{\sigma_i}{2\tau_i}\frac{\partial^2}{\partial x_i^2}\left(x^2 P(x_i, t)\right) \tag{B}$$

To solve for the stationary distribution $P^*(x_i) = \lim_{t\to\infty} P(x_i, t)$ we first set the left hand side of Eq.B to zero and rearrange

$$\left(m_i + \frac{x_i}{\tau_i}\left(1 - \frac{x_i}{K_i}\right)\right) P^*(x_i) = \frac{\sigma_i}{2\tau_i}\frac{\partial}{\partial x_i}\left(x_i^2 P^*(x_i)\right) \tag{C}$$

Following the derivation in [2], we set $x_i^2 P^*(x_i) \equiv Q(x_i)$, obtaining

$$\left(\frac{m_i}{x^2} + \frac{1}{x_i \tau_i} - \frac{1}{K_i \tau_i}\right) Q(x_i) = \frac{\sigma_i}{2\tau_i} Q^{'}(x_i) \tag{D}$$

which has the following solution

$$Q(x_i) = c \cdot \exp\left[-\frac{2}{\sigma_i x_i}\left(\tau_i m_i + \frac{x_i^2}{K_i}\right)\right] x_i^{2\sigma_i^{-1}} \tag{E}$$

where c is an arbitrary constant. Using our definition of $Q(x_i)$ and fixing c by imposing $\int_0^1 dx_i P^*(x_i) = 1$, we solve the integral

$$\int_0^\infty P^*(x_i)dx_i = \int_0^\infty c \cdot \exp\left[-\frac{2}{\sigma_i x_i}\left(\tau_i m_i + \frac{x_i^2}{K_i}\right)\right] x_i^{2\sigma_i^{-1}-2} dx_i \tag{F}$$

The solution to this integral can be solved using a known identity (Eq. 2.3.16.1 in [3]). We derived a solution for this known identity for the sake of completeness. First, to simplify the integral we define the following parameters: $p \equiv \frac{2x_i}{\sigma_i K_i}$, $q \equiv \frac{2\tau_i m_i}{\sigma_i}$, $\alpha \equiv 2\sigma_i^{-1} - 2$, and set $x_i \equiv Ce^\theta$. Using this last definition, we obtain $px_i + \frac{q}{x_i} = aCe^\theta + \frac{b}{C}e^{-\theta}$. We can then chose $pC = \frac{q}{C} \Rightarrow C = \sqrt{\frac{q}{p}}$, from which we obtain $px_i + \frac{q}{x_i} = p\sqrt{\frac{q}{p}}\left(e^\theta + e^{-\theta}\right) = 2\sqrt{pq}\cosh(\theta)$. Using this identity, we can solve the integral

$$\begin{aligned}
\int_0^\infty \exp\left[-\left(px_i + \frac{q}{x_i}\right)\right] x_i^\alpha dx_i &= \int_{-\infty}^\infty \exp\left[-2\sqrt{pq}\cosh(\theta)\right]\left(\frac{q}{p}e^\theta\right)^{\alpha+1} d\theta && \text{(Ga)}\\
&= \left(\frac{q}{p}\right)^{\frac{1}{2}(\alpha+1)} \int_{-\infty}^\infty \exp\left[-2\sqrt{pq}\cosh(\theta)\right] e^{\theta(\alpha+1)} d\theta && \text{(Gb)}\\
&= \left(\frac{q}{p}\right)^{\frac{1}{2}(\alpha+1)} \int_{-\infty}^\infty \exp\left[-2\sqrt{pq}\cosh(\theta)\right] \\
&\qquad \cdot \left[\cosh(\theta(\alpha+1)) + \sinh(\theta(\alpha+1))\right] d\theta && \text{(Gc)}\\
&= 2\left(\frac{q}{p}\right)^{\frac{1}{2}(\alpha+1)} \int_0^\infty \exp\left[-2\sqrt{pq}\cosh(\theta)\right] \\
&\qquad \cdot \cosh(\theta(\alpha+1)) d\theta && \text{(Gd)}\\
&= 2\left(\frac{q}{p}\right)^{\frac{1}{2}(\alpha+1)} B_{\alpha+1}(2\sqrt{pq}) && \text{(Ge)}
\end{aligned}$$

where B is the modified Bessel function of the second kind. After replacing our variables, we obtain a solution for the constant of integration

$$c = \left[2\left(\tau_i m_i K_i\right)^{\frac{1}{2}\left(2\sigma_i^{-1}-1\right)} \cdot B_{\left(2\sigma_i^{-1}-1\right)}\left(\frac{4}{\sigma_i}\sqrt{\frac{\tau_i m_i}{K_i}}\right)\right]^{-1} \tag{H}$$

which we use to arrive at a solution for the stationary PDF

$$P^*(x_i) = \left[2\left(\tilde{m}_i K_i\right)^{\frac{1}{2}\left(2\sigma_i^{-1}-1\right)} \cdot B_{\left(2\sigma_i^{-1}-1\right)}\left(\frac{4}{\sigma_i}\sqrt{\frac{\tilde{m}_i}{K_i}}\right)\right]^{-1} \cdot \exp\left[-\frac{2}{\sigma_i x_i}\left(\tilde{m}_i + \frac{x_i^2}{K_i}\right)\right] x_i^{2\sigma_i^{-1}-2} \tag{I}$$

which is the predicted form of the AFD with migration. We can replace the product containing the migration term with a compound parameter $\tilde{m}_i \equiv m_i \tau_i$. Using this distribution, we can then calculate the first and second moments of the PDF as

$$\begin{aligned}
\langle x_i \rangle &= \int_0^\infty P^*(x_i)\, x_i \, dx_i \\
&= \left[2\left(\tau_i m_i K_i\right)^{\frac{1}{2}\left(2\sigma_i^{-1}-1\right)} \cdot B_{\left(2\sigma_i^{-1}-1\right)}\left(\frac{4}{\sigma_i}\sqrt{\frac{\tau_i m_i}{K_i}} \right) \right]^{-1} \\
&\quad \cdot \int_0^\infty \exp\left[-\frac{2}{\sigma_i x_i}\left(\tau_i m_i + \frac{x_i^2}{K_i} \right) \right] x_i^{2\sigma_i^{-1}-1} dx_i \\
&= \left(\tau_i m_i K_i\right)^{\frac{1}{2}} \frac{B_{\left(2\sigma_i^{-1}\right)}\left(\frac{4}{\sigma_i}\sqrt{\frac{\tau_i m_i}{K_i}} \right)}{B_{\left(2\sigma_i^{-1}-1\right)}\left(\frac{4}{\sigma_i}\sqrt{\frac{\tau_i m_i}{K_i}} \right)}
\end{aligned}$$

$$\begin{aligned}
\left\langle x_i^2 \right\rangle &= \int_0^\infty P^*(x_i)\, x_i^2 \, dx_i \\
&= \left[2\left(\tau_i m_i K_i\right)^{\frac{1}{2}\left(2\sigma_i^{-1}-1\right)} \cdot B_{\left(2\sigma_i^{-1}-1\right)}\left(\frac{4}{\sigma_i}\sqrt{\frac{\tau_i m_i}{K_i}} \right) \right]^{-1} \\
&\quad \cdot \int_0^\infty \exp\left[-\frac{2}{\sigma_i x_i}\left(\tau_i m_i + \frac{x_i^2}{K_i} \right) \right] x_i^{2\sigma_i^{-1}} dx_i \\
&= \tau_i m_i K_i \frac{B_{\left(2\sigma_i^{-1}+1\right)}\left(\frac{4}{\sigma_i}\sqrt{\frac{\tau_i m_i}{K_i}} \right)}{B_{\left(2\sigma_i^{-1}-1\right)}\left(\frac{4}{\sigma_i}\sqrt{\frac{\tau_i m_i}{K_i}} \right)}
\end{aligned}$$

From which we can derive the squared coefficient of variation

$$\frac{\left\langle x_i^2 \right\rangle - \left\langle x_i \right\rangle^2}{\left\langle x_i \right\rangle^2} = \left(\frac{B_{\left(2\sigma_i^{-1}+1\right)}\left(\frac{4}{\sigma_i}\sqrt{\frac{\tau_i m_i}{K_i}} \right)}{B_{\left(2\sigma_i^{-1}\right)}\left(\frac{4}{\sigma_i}\sqrt{\frac{\tau_i m_i}{K_i}} \right)} \right)^2 - 1 \qquad \text{(J)}$$

We ultimately want to test the feasibility of the sampling distribution, which we define as the convolution of the AFD and the probability of sampling n^{reads} for a given species from a Poisson distribution out of N^{reads} total reads.

$$\begin{aligned}
P(n_i | K_i, \sigma_i, \tilde{m}_i, N) &= \int_0^\infty P^*(x_i | K_i, \sigma_i, \tilde{m}_i) P(n_i | N, x_i)\, dx_i \\
&= \left[2\left(\tilde{m}_i K_i\right)^{\frac{1}{2}\left(2\sigma_i^{-1}-1\right)} \cdot B_{\left(2\sigma_i^{-1}-1\right)}\left(\frac{4}{\sigma_i}\sqrt{\frac{\tilde{m}_i}{K_i}} \right) \right]^{-1} \\
&\quad \cdot \frac{N^{n_i}}{n_i!} \int_0^\infty \exp\left[-\left(\frac{2\tilde{m}_i}{\sigma_i x_i} + \frac{2x_i}{\sigma_i K_i} + N x_i \right) \right] x^{2\sigma_i^{-1}-2+n_i} e^{-N x_i} dx_i \\
&= \frac{N^{n_i}}{n_i!} \left(\tilde{m}_i K_i\right)^{-\frac{1}{2}\left(2\sigma_i^{-1}-1\right)} \left(\frac{2\tilde{m}_i K_i}{2 + N\sigma_i K_i} \right)^{\frac{1}{2}\left(2\sigma^{-1}-1+n_i\right)} \\
&\quad \cdot \frac{B_{\left(2\sigma_i^{-1}-1+n_i\right)}\left(\sqrt{\frac{8\tilde{m}_i}{\sigma_i}\left(\frac{2}{\sigma_i K_i} + N \right)} \right)}{B_{\left(2\sigma_i^{-1}-1\right)}\left(\frac{4}{\sigma_i}\sqrt{\frac{\tilde{m}_i}{K_i}} \right)}
\end{aligned}$$

While the terms describing the mean and squared CV are correct, they are unwieldy. To make progress, it is useful to reduce Eq. I at different parameter limits to examine

its behavior as well as how it differs from the stationary PDF of the SLM in the absence of migration (Eq. C). We start be identifying limiting forms of the Bessel function. In the limit $B_v(y) \sim \frac{1}{2}\Gamma(v)\left(\frac{y}{2}\right)^{-v}$ as $y \to 0$, which corresponds to $\tilde{m}_i \ll \left(\frac{4}{\sigma_i}\right)^2 K_i$, the moments of the distribution reduce to those obtained from the SLM without migration.

Contrastingly, as $y \to \infty$ the Bessel function can be approximated by the asymptotic expansion $B_v(y) \sim \sqrt{\frac{\pi}{2y}} e^{-y}\left[1 + \frac{4v^2-1}{8y} + \frac{\left(4v^2-1\right)\left(4v^2-9\right)}{2!(8y)^2} + \cdots\right]$, which corresponds to the high migration limit $\tilde{m}_i \gg \left(\frac{4}{\sigma_i}\right)^2 K_i$. Using the first term of this expansion, we arrive at the stationary PDF.

$$P^*(x_i) = \sqrt{\frac{8}{\pi\sigma_i}}\left(\frac{\tau_i m_i}{K_i}\right)^{\frac{1}{4}} \cdot \exp\left[\frac{2}{\sigma_i}\left(2\sqrt{\frac{\pi m_i}{K_i}} - \frac{\tau_i m_i}{x_i} - \frac{x_i}{K_i}\right)\right] x_i^{2\sigma^{-1}-1} \quad \text{(K)}$$

This limiting form of the stationary PDF is, again, unwieldy, but it is clear from visual inspection that it does not resemble the gamma distribution. By taking the same limit of the moments of the AFD with migration, we obtain the following approximations

$$\begin{aligned} \langle x_i \rangle &\approx \sqrt{\tilde{m}_i K_i}\frac{\frac{32}{\sigma}\sqrt{\frac{\tilde{m}_i}{K_i}} + 4(\frac{2}{\sigma})^2 - 1}{\frac{32}{\sigma}\sqrt{\frac{\tilde{m}_i}{K_i}} + 4(\frac{2}{\sigma} - 1)^2 - 1} \\ \frac{\langle x_i^2 \rangle - \langle x_i \rangle^2}{\langle x_i \rangle^2} &\approx \left(\frac{1 + \frac{4(2\sigma_i^{-1}+1)^2-1}{\frac{32}{\sigma_i}\sqrt{\frac{\tilde{m}_i}{K_i}}}}{1 + \frac{4(2\sigma_i^{-1})^2-1}{\frac{32}{\sigma_i}\sqrt{\frac{\tilde{m}_i}{K_i}}}}\right)^2 - 1 \end{aligned} \quad \text{(L)}$$

References

1. C. W. Gardiner and C. W. Gardiner. *Stochastic methods: a handbook for the natural and social sciences*. Springer series in synergetics. Springer, Berlin, 4th ed edition, 2009.

2. Jacopo Grilli. Macroecological laws describe variation and diversity in microbial communities. *Nature Communications*, 11(1):4743, September 2020. Bandiera_abtest: a Cc_license_type: cc_by Cg_type: Nature Research Journals Number: 1 Primary_atype: Research Publisher: Nature Publishing Group Subject_term: Biodiversity;Community ecology;Ecological modelling;Macroecology;Microbial ecology Subject_term_id: biodiversity;community-ecology;ecological-modelling;macroecology;microbial-ecology.

3. Anatolij P. Prudnikov, Jurij A. Bryčkov, and Oleg I. Maričev. *Integrals and series. 1: Elementary functions*. Gordon and Breach Science Publ, New York, NY, 4. print edition, 1998.
