# Supplementary material for: Macroecological patterns in experimental microbial communities
Source: PLoS Comput Biol. 2025 May 8;21(5):e1013044. doi: 10.1371/journal.pcbi.1013044 (PMC12112161; doi:10.1371/journal.pcbi.1013044)
Supplement: S5 Text — The time-dependent probability distribution of abundance for the SLM. (PDF) [file pcbi.1013044.s005.pdf]

Macroecological patterns in experimental microbial communities: S5 Text

William R. Shoemaker[1,*], Álvaro Sánchez[2], and Jacopo Grilli[1]

1 Quantitative Life Sciences, The Abdus Salam International Centre for Theoretical Physics (ICTP), Trieste, 34151, Italy.
2 Instituto de Biología Funcional y Genómica, IBFG-CSIC, Universidad de Salamanca, 37007, Salamanca, Spain.

* **Contact:** williamrshoemaker@gmail.com

S5 Text: Obtaining the time-dependent AFD for the SLM

To contrast the effect of a constant rate of migration (i.e., chemostat) and migration as an initial condition (i.e., batch culture) we used the time-dependent probability distribution of abundances for the SLM with no migration ($P(x,t|x_0)$). To our knowledge, this solution was first derived by Schenzle and Brand and later rederived by Otunuga [1,2]. Using the latter derivation, the time-dependent distribution is

$$\begin{aligned} P(x,t|x_0) = x^{2\sigma_i^{-1}-1} e^{-\frac{2x}{K_i\sigma_i}} \sum_{m=0}^{M} \Bigg[\left(\frac{2}{K_i\sigma_i}\right)^{v_m} \frac{m! v_m}{\Gamma(v_m+m+1)} e^{-\lambda_n t} (x * x_0)^{-m} \\ \cdot L_m^{v_m}\left(\frac{2x}{K_i\sigma_i}\right) L_m^{v_m}\left(\frac{2x_0}{K_i\sigma_i}\right)\Bigg] \\ + A x^{2\sigma_i^{-1}-1} e^{-\frac{2x}{K_i\sigma_i}} \\ \cdot \int_0^\infty e^{-\lambda(\eta)t} h(\eta, x_0^{-1}) h(\eta, x^{-1}) d\eta \end{aligned} \tag{A}$$

where $L_m^{v_m}$ is the Laguerre polynomial of degree m and the summation occurs over the range $\sigma_i^{-1} - \frac{3}{2} \leq M \leq \sigma_i^{-1} - \frac{1}{2}$. The remaining terms of the function are

$$\lambda_m = \frac{m\sigma_i}{2\tau_i}\left(2\sigma_i^{-1} - 1 - \eta\right) \tag{Ba}$$

$$\lambda(\eta) = \frac{\sigma_i}{2\tau_i}\left(\left(\sigma_i^{-1} - \frac{1}{2}\right) + \eta^2\right) \tag{Bb}$$

$$h(\eta, y) = G_\eta y^{\sigma_i^{-1}} e^{\frac{1}{K_i\sigma_i y}} W_{\sigma_i^{-1}, i\eta}\left(\frac{1}{K_i\sigma_i y}\right) \tag{Bc}$$

$$G_\eta = \sqrt{\frac{\sigma_i K_i}{2\pi^2 A}\eta \sinh(2\pi\eta)\Gamma\left(\frac{1}{2} - \sigma_i^{-1} + i\eta\right)\Gamma\left(\frac{1}{2} - \sigma_i^{-1} - i\eta\right)} \tag{Bd}$$

$$v_m = 2\sigma_i^{-1} - 2m \tag{Be}$$

$$A = \left[\left(\frac{\sigma_i K_i}{2}\right)^{2\sigma_i^{-1}-1} \Gamma\left(2\sigma_i^{-1} - 1\right)\right]^{-1} \tag{Bf}$$

where $W_{\kappa,i\eta}\left(\cdot\right)$ is the second solution to the Whittaker differential equation. To briefly summarize how this solution was obtained, the solution to the FPE was assumed to follow an eigenvalue form, from which a Kolmogorov Backward Equation was obtained. Using the stationary solution, a differential equation was obtained that reduced to the Kummer differential equation, from which a solution was obtained. Here we have used the solution of the Itô form of the SLM with migration, consistent with our derivation in S1.

The time-dependent mean and variance could then be derived from Eq. A to evaluate how the exponent of Taylor's Law is shaped by the length of time that has transpired since the start of the transfer cycle (t) and the initial abundance due to migration (x_0). However, given the form of the time-dependent solution, these functions are unwieldy. Therefore, we elected to numerically simulate the SLM to determine how the exponent of Taylor's Law changed before and after the cessation of migration manipulations.

References

1. A. Schenzle and H. Brand. Multiplicative stochastic processes in statistical physics. *Physical Review A*, 20(4):1628–1647, October 1979.

2. Olusegun Michael Otunuga. Time-dependent probability density function for general stochastic logistic population model with harvesting effort. *Physica A: Statistical Mechanics and its Applications*, 573:125931, July 2021.
