# Supplementary material for: Macroecological patterns in experimental microbial communities
Source: PLoS Comput Biol. 2025 May 8;21(5):e1013044. doi: 10.1371/journal.pcbi.1013044 (PMC12112161; doi:10.1371/journal.pcbi.1013044)
Supplement: S6 Text — Additional details and derivations related to our SLM simulations. (PDF) [file pcbi.1013044.s006.pdf]

Macroecological patterns in experimental microbial communities: S6 Text

William R. Shoemaker[1,*], Álvaro Sánchez[2], and Jacopo Grilli[1]

1 Quantitative Life Sciences, The Abdus Salam International Centre for Theoretical Physics (ICTP), Trieste, 34151, Italy.
2 Instituto de Biología Funcional y Genómica, IBFG-CSIC, Universidad de Salamanca, 37007, Salamanca, Spain.

*** Contact:** `williamrshoemaker@gmail.com`

S6 Text: Simulating the SLM

Here we describe how sampling, both in terms of inoculation as well as sequencing, was implements in our SLM simulations as well as how the SLM simulations were performed. The process of sampling community members at the end of a transfer cycle can be modeled as multinomial sampling process, where we obtain a vector of initial abundances of all ASVs $\vec{n}^{(k)}(0)$

$$\Pr\left[\vec{n}^{(k)}(0)\right] = \begin{cases} \frac{N_{\mathrm{inoc}}!}{n_1!\cdots n_S!} x_{1,\mathrm{progenitor}}^{n_1} \cdots x_{S,\mathrm{progenitor}}^{n_S}, & \text{if } k = 0 \\ \frac{N^{(k)}(0)!}{n_1!\cdots n_S!} (x_1^{(k-1)}(T))^{n_1} \cdots (x_S^{(k-1)}(T))^{n_S}, & \text{if } k > 0 \end{cases} \tag{A}$$

The vector of S ASV abundances at the start of the kth transfer cycle ($\vec{n}^{(k)}(0)$) are drawn from the progenitor community for the first transfer cycle ($k = 0$) and are then drawn from the previous transfer at $T = 48$ h. for all subsequent transfer cycles ($k > 0$). The term N_{inoc} represents the number of individuals sampled from the progenitor community when each replicate community was initiated.

Similarly, the manipulation of abundances at the start of a transfer cycle due to migration can be modeled as a multinomial sampling process

$$\Pr\left[\vec{n}^{(k)}_{\mathrm{migration}}\right] = \begin{cases} \frac{N_{\mathrm{regional}}!}{n_1!\cdots n_S!} x_{1,\mathrm{progenitor}}^{n_1} \cdots x_{S,\mathrm{progenitor}}^{n_S}, & \text{Regional, } k \leq 12 \\ \frac{N_{\mathrm{global}}!}{n_1!\cdots n_S!} (x_{1,\mathrm{global}}^{(k-1)})^{n_1} \cdots (x_{S,\mathrm{global}}^{(k-1)})^{n_S}, & \text{Global, } k \leq 12 \end{cases} \tag{B}$$

where $\vec{n}^{(k)}_{\mathrm{migration}}(0) = \vec{0}$ for $k > 12$ since at this point the migration manipulations have ceased.

Beyond migration, there are dependencies between the final abundance of an ASV and its abundance in the progenitor community that we would like to incorporate into our model. The set of carrying capacities K_i was drawn from the parameters obtained by fitting Eq. 9 to the empirical MAD. The dependence between abundances in the progenitor community $\vec{x}_{\mathrm{progenitor}}$ and the empirical MAD were evaluated by performing logistic regression from `scikit-learn v0.22.1` [1].

Initially, the weak correlations observed between the mean relative abundance after the cessation of migration ($\bar{x}_i(t = 18)$) and the relative abundance observed in the

progenitor ($x_{i,\text{progenitor}}$) would imply that the carrying capacity of a given ASV in the assembled communities was independent of its abundance in the progenitor community (Fig S4). However, this observation suffers from survivorship bias, since we can only compare values of $\bar{x}_i$ and $x_{i,\text{progenitor}}$ for ASVs that were actually present in both the progenitor and assembled communities (i.e., ASVs with $K_i > 0$). A permutational Kolmogorov–Smirnov test found that this shift was significant, a result that held for all experimental treatments (Fig S6). We modeled the dependency between the occupancy of an ASV is non-zero and its abundance in the progenitor using logistic regression as follows (Fig S5b):

$$\Pr[K_i > 0 | x_{i,\text{progenitor}}] \propto [1 + \exp(-(a + b * \log_{10} x_{i,\text{progenitor}}))]^{-1} \tag{C}$$

where a is the intercept and b is the slope of the regression.

From this relationship we can model the probability that an ASV has a non-zero carrying capacity $\Pr[K_i > 0 | x_{i,\text{progenitor}}]$, allowing us to define the full distribution of carrying capacities as follows

$$K_i \sim \begin{cases} \text{Lognorm} & \text{with probability } \Pr[K_i > 0 | x_{i,\text{progenitor}}] \\ 0 & \text{with probability } 1 - \Pr[K_i > 0 | x_{i,\text{progenitor}}] \end{cases} \tag{D}$$

The total number of ASVs with $K_i > 0$ was drawn from the distribution $S_{\text{descendant}} \sim \text{Binomial}(S_{\text{progenitor}}, 0.016)$, where $S_{\text{progenitor}}$ is the number of ASVs in the progenitor community and 0.016 is the mean fraction of ASVs that were present in the assembled communities relative to the number of ASVs in the progenitor community.

Grafting these empirical considerations onto the time-dependent analytic solution of the SLM is not straightforward. Therefore, we elected to simulate the SLM. We used an approximation of the numerical solution of the SLM for the dynamics within each transfer cycle, incorporating above experimental details while requiring only two parameters: σ and τ, the values of the latter being constrained by the amount of growth that can possibly occur over 48 h. within a transfer cycle. To simulate the SLM, we derived a form of Eq. 1 so that the noise was additive instead of multiplicative by introducing the change of variable $q_i = \log(x_i)$, expanding dq_i as a Taylor series, and using Itô's lemma.

$$dq_i = \frac{\partial q_i}{\partial t} dt + \frac{\partial q_i}{\partial x_i} dx_i + \frac{1}{2} \frac{\partial^2 q_i}{\partial x_i^2} (dx_i)^2 \tag{Ea}$$

$$= \frac{\partial q_i}{\partial t} dt + \frac{\partial q_i}{\partial x_i} \left(\frac{x_i}{\tau_i} \left(1 - \frac{x_i}{K_i}\right) dt + \sqrt{\frac{\sigma_i}{\tau_i}} x_i dW(t) \right) + \frac{1}{2} \frac{\partial^2 q_i}{\partial x_i^2} \left(\frac{x_i}{\tau_i} \left(1 - \frac{x_i}{K_i}\right) dt + \sqrt{\frac{\sigma_i}{\tau_i}} x_i dW(t) \right)^2 \tag{Eb}$$

$$= \left(\frac{\partial q_i}{\partial t} + \frac{\partial q_i}{\partial x_i} \frac{x_i}{\tau_i} \left(1 - \frac{x_i}{K_i}\right) + \frac{\sigma_i}{2\tau_i} \frac{\partial^2 q_i}{\partial x_i^2} x_i^2 \right) dt + \sqrt{\frac{\sigma_i}{\tau_i}} \frac{\partial q_i}{\partial x_i} x_i dW(t) \tag{Ec}$$

where $W(t)$ is a Wiener process. We then obtain the Langevin by dividing both sides by dt

$$\frac{dq_i}{dt} = \frac{1}{\tau_i} \left(1 - \frac{\sigma_i}{2} - \frac{e^{q_i}}{K_i}\right) + \sqrt{\frac{\sigma_i}{\tau_i}} \eta(t) \tag{F}$$

We can approximate the numerical solution to Eq. F using the Euler–Maruyama method [2], where we simulate the discretized form of the equation, defining δt as a single generation.

$$q_i(t+\delta t) = q_i(t) + \frac{1}{\tau_i}\left(1 - \frac{\sigma_i}{2} - \frac{e^{q_i(t)}}{K_i}\right)\delta t + \sqrt{\frac{\sigma_i \delta t}{\tau_i}} Z(t) \tag{G}$$

where $Z(t) \sim \mathcal{N}(0,1)$. We set σ as a constant due to the existence of Taylor's Law and τ as a constant due to the unknown nature of the distribution of growth rates. We used logarithmically spaced parameter values from the following ranges: $\sigma \in [0.01, 1.9]$ and $\tau \in [1.7, 6.9]$. The upper bound on σ was determined by the mathematical requirement that $\sigma < 2$. The range on τ was set by the observation that the serial dilution factor sets the total number of generations per-transfer as $\log_2(D_{\text{transfer}}^{-1}) \sim 7$, assuming exponential growth. This translates to a maximum generation time of $\tau_{\text{max}} = 48\text{h.}/7 \sim 6.9\text{h}$. We identified a reasonable bound on the minimum generation time by noticing that prior research efforts have established that these communities have a maximum growth rate of $\sim 0.6\,\text{h}^{-1}$, translating to a minimum generation time of $\tau_{\text{min}} \equiv 0.6^{-1} \approx 1.7\text{h}$ [3].

Simulated relative abundances were calculated from simulated true abundances, with the sampling process modeled as a multinomial process

$$\Pr\left[\vec{n}_{\text{reads}}^{(k)}\right] = \frac{N_{\text{reads}}!}{n_1! \cdots n_S!}(x_1^{(k)})^{n_1} \cdots (x_S^{(k)})^{n_S} \tag{H}$$

The empirical total number of reads N_{reads} was used for all simulations (see Fig S7 for visualization of the variation in sampling effort). Information about the statistical analysis can be found in the Materials and Methods.

References

1. Fabian Pedregosa, Gaël Varoquaux, Alexandre Gramfort, Vincent Michel, Bertrand Thirion, Olivier Grisel, Mathieu Blondel, Peter Prettenhofer, Ron Weiss, Vincent Dubourg, Jake Vanderplas, Alexandre Passos, David Cournapeau, Matthieu Brucher, Matthieu Perrot, and Édouard Duchesnay. Scikit-learn: Machine Learning in Python. *Journal of Machine Learning Research*, 12(85):2825–2830, 2011.

2. C. W. Gardiner and C. W. Gardiner. *Stochastic methods: a handbook for the natural and social sciences*. Springer series in synergetics. Springer, Berlin, 4th ed edition, 2009.

3. Sylvie Estrela, Jean C. C. Vila, Nanxi Lu, Djordje Bajić, Maria Rebolleda-Gómez, Chang-Yu Chang, Joshua E. Goldford, Alicia Sanchez-Gorostiaga, and Alvaro Sanchez. Functional attractors in microbial community assembly. *Cell Systems*, 13(1):29–42.e7, January 2022.
