# Supplementary material for: Macroecological patterns in experimental microbial communities
Source: PLoS Comput Biol. 2025 May 8;21(5):e1013044. doi: 10.1371/journal.pcbi.1013044 (PMC12112161; doi:10.1371/journal.pcbi.1013044)
Supplement: S7 Text — Derivations of the effect of global migration on moments of abundance in a batch culture experimental design framework. (PDF) [file pcbi.1013044.s007.pdf]

Macroecological patterns in experimental microbial communities: S7 Text

William R. Shoemaker[1,*], Álvaro Sánchez[2], and Jacopo Grilli[1]

1 Quantitative Life Sciences, The Abdus Salam International Centre for Theoretical Physics (ICTP), Trieste, 34151, Italy.
2 Instituto de Biología Funcional y Genómica, IBFG-CSIC, Universidad de Salamanca, 37007, Salamanca, Spain.

*** Contact:** `williamrshoemaker@gmail.com`

S7 Text: Effect of global migration on the statistical moments of abundance

The prediction that global migration would decrease fluctuations in abundance while leaving the expected value unchanged can be justified based on experimental details and the properties of statistical moments of distributions. To perform the global migration treatment, aliquots were taken of each replicate community on the $k-1$th transfer cycle after T hours, pooled together and intermixed, then redistributed among the same communities at the start of the kth transfer cycle (Fig 3). Ignoring fluctuations driven by sampling, the relative abundance of an ASV in the intermixed pool before it is redistributed will be $x_{i,\mathrm{global}}^{(k-1)} \equiv \frac{1}{M}\sum_{m}^{M} x_{i,m}^{(k-1)}(T)$, where the M^{-1} prefactor accounts for an equal volume of each aliquot having been sampled. From this definition, we obtain the expected relative abundance in the global migration pool

$$\left\langle x_{i,\mathrm{global}}^{(k-1)} \right\rangle = \left\langle \frac{1}{M}\sum_{m}^{M} x_{i}^{(k-1)}(T) \right\rangle \tag{Aa}$$

$$= \left\langle x_{i}^{(k-1)}(T) \right\rangle \tag{Ab}$$

and the variance of relative abundance in the global migration pool

$$\mathrm{Var}\left(x_{i,\mathrm{global}}^{(k-1)} \right) = \mathrm{Var}\left(\frac{1}{M}\sum_{m}^{M} x_{i}^{(k-1)}(T) \right) \tag{Ba}$$

$$= \frac{1}{M^2}\sum_{m}^{M} \mathrm{Var}\left(x_{i}^{(k-1)}(T) \right) \tag{Bb}$$

$$= \frac{1}{M}\mathrm{Var}\left(x_{i}^{(k-1)}(T) \right) \tag{Bc}$$

When an aliquot of the intermixed global migration pool is sampled and added to a community at the start of transfer cycle k, we find that the mean relative abundance at

the start of a transfer cycle remains unchanged

$$\left\langle x_i^{(k)}(0) \right\rangle = \left\langle \frac{D_{\mathrm{transfer}} x_i^{(k-1)}(T) N^*(T) + D_{\mathrm{global}} x_{i,\mathrm{global}}^{(k-1)} N_{\mathrm{global}}}{D_{\mathrm{transfer}} N^*(T) + D_{\mathrm{global}} N_{\mathrm{global}}} \right\rangle \tag{Ca}$$

$$= \left\langle x_i^{(k-1)}(T) \right\rangle \frac{D_{\mathrm{transfer}} N^*(T) + D_{\mathrm{global}} N_{\mathrm{global}}}{D_{\mathrm{transfer}} N^*(T) + D_{\mathrm{global}} N_{\mathrm{global}}} \tag{Cb}$$

$$= \left\langle x_i^{(k-1)}(T) \right\rangle \tag{Cc}$$

In contrast, when deriving the variance as fluctuations around the expected relative abundance, we obtain the following inequality

$$\mathrm{Var}\left(x_i^{(k)}(0)\right) = \frac{(D_{\mathrm{transfer}} N^*(T))^2 \mathrm{Var}\left(x_i^{(k-1)}(T)\right) + (D_{\mathrm{global}} N_{\mathrm{global}})^2 \mathrm{Var}\left(x_{i,\mathrm{global}}^{(k-1)}\right)}{(D_{\mathrm{transfer}} N^*(T) + D_{\mathrm{global}} N_{\mathrm{global}})^2} \tag{Da}$$

$$= \frac{(D_{\mathrm{transfer}} N^*(T))^2 \mathrm{Var}\left(x_i^{(k-1)}(T)\right) + (D_{\mathrm{global}} N_{\mathrm{global}})^2 \frac{1}{M} \mathrm{Var}\left(x_i^{(k-1)}(T)\right)}{(D_{\mathrm{transfer}} N^*(T) + D_{\mathrm{global}} N_{\mathrm{global}})^2} \tag{Db}$$

$$= \mathrm{Var}\left(x_i^{(k-1)}(T)\right) \frac{(D_{\mathrm{transfer}} N^*(T))^2 + (D_{\mathrm{global}} N_{\mathrm{global}})^2 \frac{1}{M}}{(D_{\mathrm{transfer}} N^*(T) + D_{\mathrm{global}} N_{\mathrm{global}})^2} \tag{Dc}$$

$$< \mathrm{Var}\left(x_i^{(k-1)}(T)\right) \tag{Dd}$$

Therefore, under the SLM we expect global migration to alter the CV of relative abundances while leaving the mean unchanged.
