# Supplementary material for: Macroecological patterns in experimental microbial communities
Source: PLoS Comput Biol. 2025 May 8;21(5):e1013044. doi: 10.1371/journal.pcbi.1013044 (PMC12112161; doi:10.1371/journal.pcbi.1013044)
Supplement: S8 Text — Additional details about SLM parameter inference. (PDF) [file pcbi.1013044.s008.pdf]

Macroecological patterns in experimental microbial communities: S8 Text

William R. Shoemaker[1,*], Álvaro Sánchez[2], and Jacopo Grilli[1]

1 Quantitative Life Sciences, The Abdus Salam International Centre for Theoretical Physics (ICTP), Trieste, 34151, Italy.
2 Instituto de Biología Funcional y Genómica, IBFG-CSIC, Universidad de Salamanca, 37007, Salamanca, Spain.

*** Contact:** `williamrshoemaker@gmail.com`

S8 Text: Inferring SLM parameters

Parameter combinations of τ, σ that produced simulated statistics which most closely matched statistics estimated from the experimental data were identified using Approximate Bayesian Computation. For analyses with a single statistic we selected the simulation iteration with the lowest Euclidean distance between the observed and simulated statistic. For analyses where a set containing multiple test statistics ($\vec{c}$) were considered, we used a weighted measure of Euclidean distance where the simulated values of each test statistic were scaled by their standard deviation to prevent the distance from being dominated by a single statistic with high variance [1].

$$d(\vec{c}_{\mathrm{obs}}, \vec{c}_{\mathrm{sim}}) = \left[\sum_{g=1}^{G} \left(\frac{c_{\mathrm{sim}}^{(g)} - c_{\mathrm{obs}}^{(g)}}{\sqrt{\mathrm{Var}(c_{\mathrm{sim}}^{(g)})}} \right)^2 \right]^{\frac{1}{2}} \tag{A}$$

This procedure was only used to identify parameters for the regional migration statistics and for the global migration $\Delta \ell$ statistics. Once the selected values of τ, σ were identified they were used to perform 10^3 simulations of our SLM model, generating a distribution of predicted summary statistics that was compared to the observed summary statistic.

Our rationale for using ABC was that it provided the flexibility necessary to investigate the properties of microbial communities, where the variation one observes can be modeled as an outcome of a disordered system (i.e., parameters such as carrying capacity being drawn from a specified distribution [2]) and 2) is in-part driven by the inherent stochasticity of sampling. This flexibility comes with the constraint that the effectiveness of ABC can be limited by the availability of informative statistics for a given model [3, 4]. In addition, the number of summary statistics presents its own issues, as it increases the dimensionality of the sampling space, resulting in an exponential decline in the probability of accepting a given simulation under ABC criteria [5]. However, it is unlikely that these issues shaped the results of this study. Whenever possible, we elected to examine patterns and quantities that are frequently used and tested in macroecological studies. When it was necessary to identify additional statistics to evaluate the macroecological outcomes of specific treatments, we scoured the literature for statistical tests that account for the standard error of an estimator (e.g., Fisher's Z-statistic for the difference in correlation coefficients, Eq. 13). Dimensionality

is also unlikely to be a major contributor, as we used at most two summary statistics to infer our two parameters. However, given that the identification of treatment-specific statistics was vital for testing the effects of specific forms of migration, it is apparent that the appropriate summary statistic(s) for a given experiment are not always obvious *a priori* and that investigating a variety of summary statistics is a worthwhile effort if one chooses to proceed with ABC.

References

1. Dennis Prangle. Adapting the ABC Distance Function. *Bayesian Analysis*, 12(1), March 2017.

2. Madhu Advani, Guy Bunin, and Pankaj Mehta. Statistical physics of community ecology: a cavity solution to MacArthur's consumer resource model. *Journal of Statistical Mechanics: Theory and Experiment*, 2018(3):033406, March 2018. Publisher: IOP Publishing and SISSA.

3. Jody Hey and Carlos A. Machado. The study of structured populations — new hope for a difficult and divided science. *Nature Reviews Genetics*, 4(7):535–543, July 2003. Number: 7 Publisher: Nature Publishing Group.

4. Katalin Csilléry, Michael G. B. Blum, Oscar E. Gaggiotti, and Olivier François. Approximate Bayesian Computation (ABC) in practice. *Trends in Ecology & Evolution*, 25(7):410–418, July 2010.

5. Mark A. Beaumont, Wenyang Zhang, and David J. Balding. Approximate Bayesian computation in population genetics. *Genetics*, 162(4):2025–2035, December 2002.
