# Supplementary figures and images for: Macroecological patterns in experimental microbial communities
Source: PLoS Comput Biol. 2025 May 8;21(5):e1013044. doi: 10.1371/journal.pcbi.1013044 (PMC12112161; doi:10.1371/journal.pcbi.1013044)

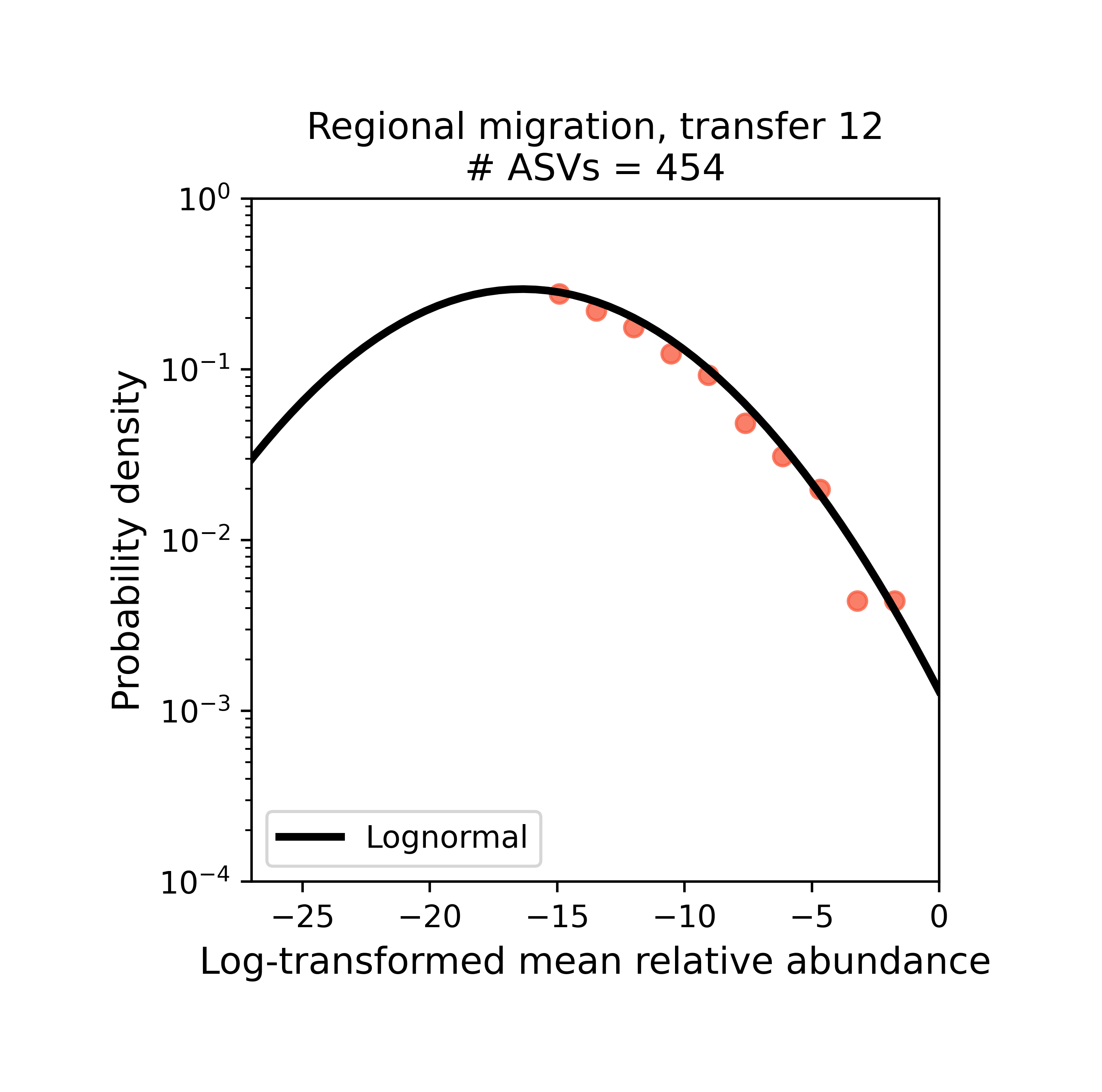

Supplement: S2 Fig — In Fig 2d the MAD of each treatment were separately rescaled to facilitate comparison, meaning that a single lognormal was fit. The lognormal parameters (μ and s) slightly vary from treatment-to-treatment, so lognormal fits were examined for separate treatments. Here we present the MAD with fitted lognormal for the regional migration treatment at transfer 12, the treatment with the highest number of ASVs. We see that the empirical MAD is non-linear on a log-log scale, suggesting that a power law would not serve as an appropriate descriptor. (TIFF) [file pcbi.1013044.s010.tiff]

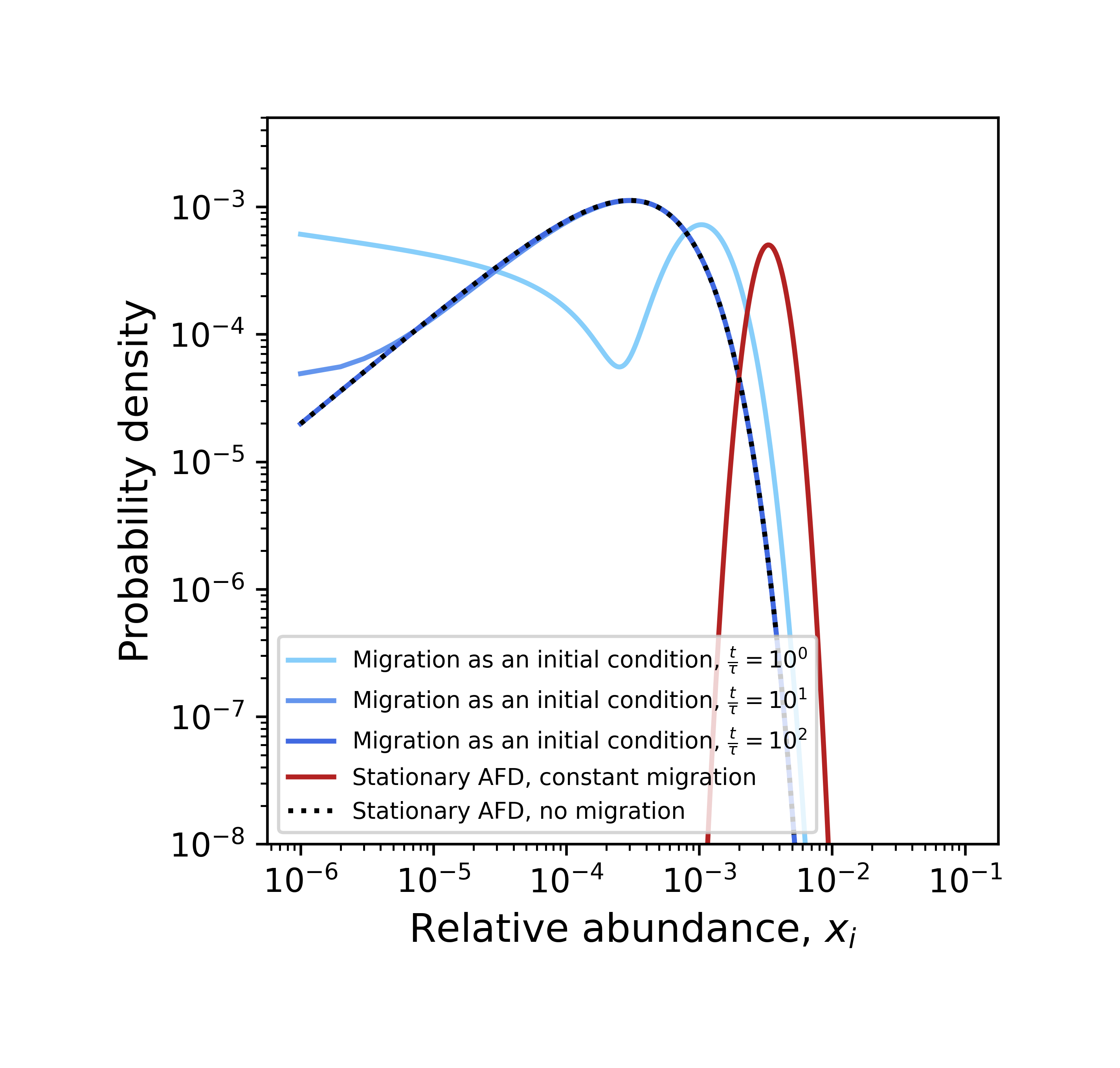

Supplement: S3 Fig — We examined how migration as a perturbation of initial conditions compared to a commonly assumed form of migration where it occurs at a constant rate per-unit time. The AFD of a form of the SLM with a constant rate of migration at stationarity was derived (S4 Text) and the time-dependent solution of the SLM was obtained from a prior study (S5 Text) . The following parameters were used: Ki=10−3, σi=0.7, τ=1, and xi(0)=mi=10−2. We have rescaled time using the timescale of growth to arrive at a dimensionless parameter tτ. The AFD with no experimentally-imposed migration is represented by Eq 2 (i.e., a gamma distribution). (TIFF) [file pcbi.1013044.s011.tiff]

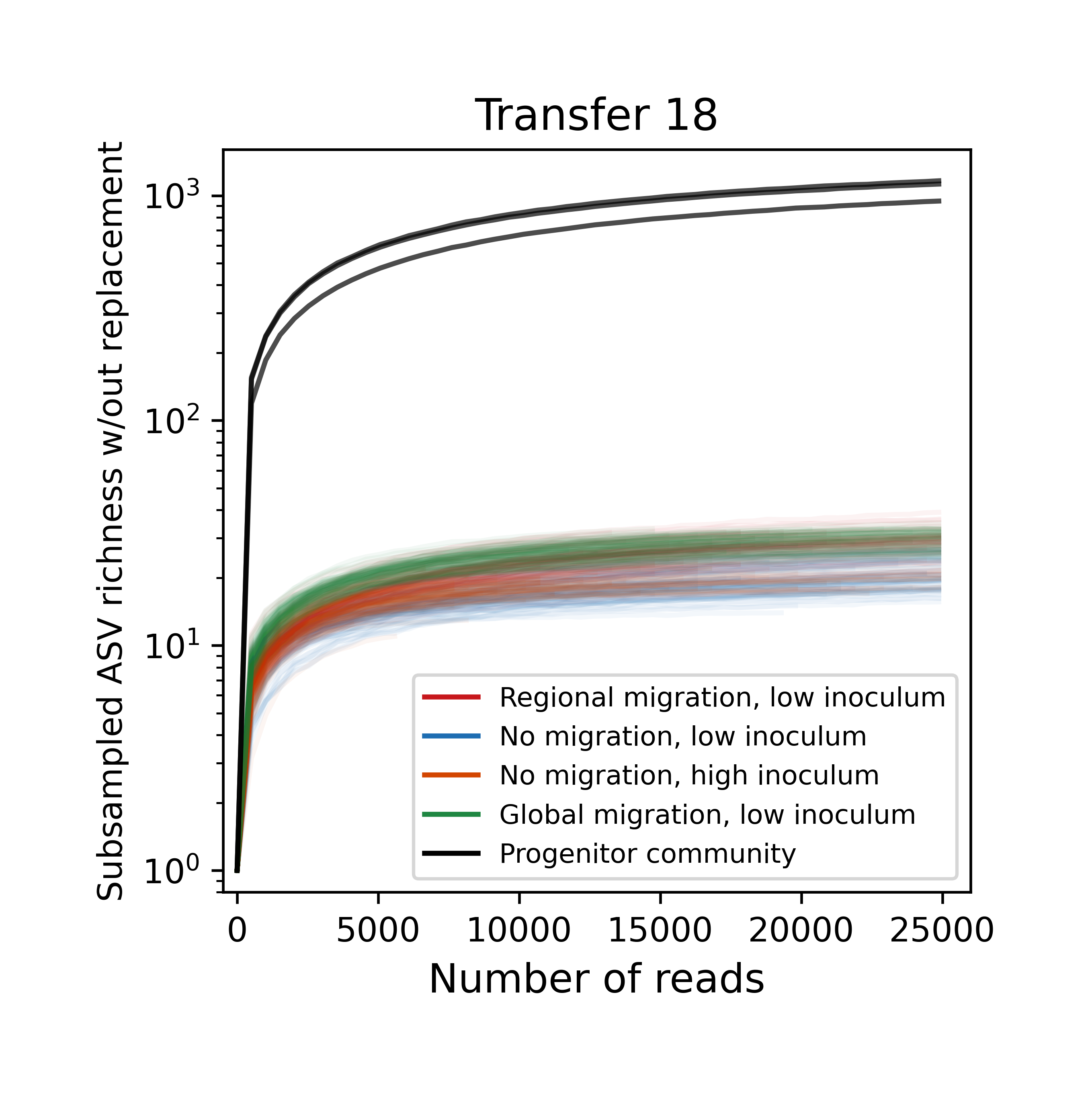

Supplement: S4 Fig — Rarefaction curves demonstrate how the richness (# ASVs) is 100-fold lower in descendant communities relative to the progenitor. (TIFF) [file pcbi.1013044.s012.tiff]

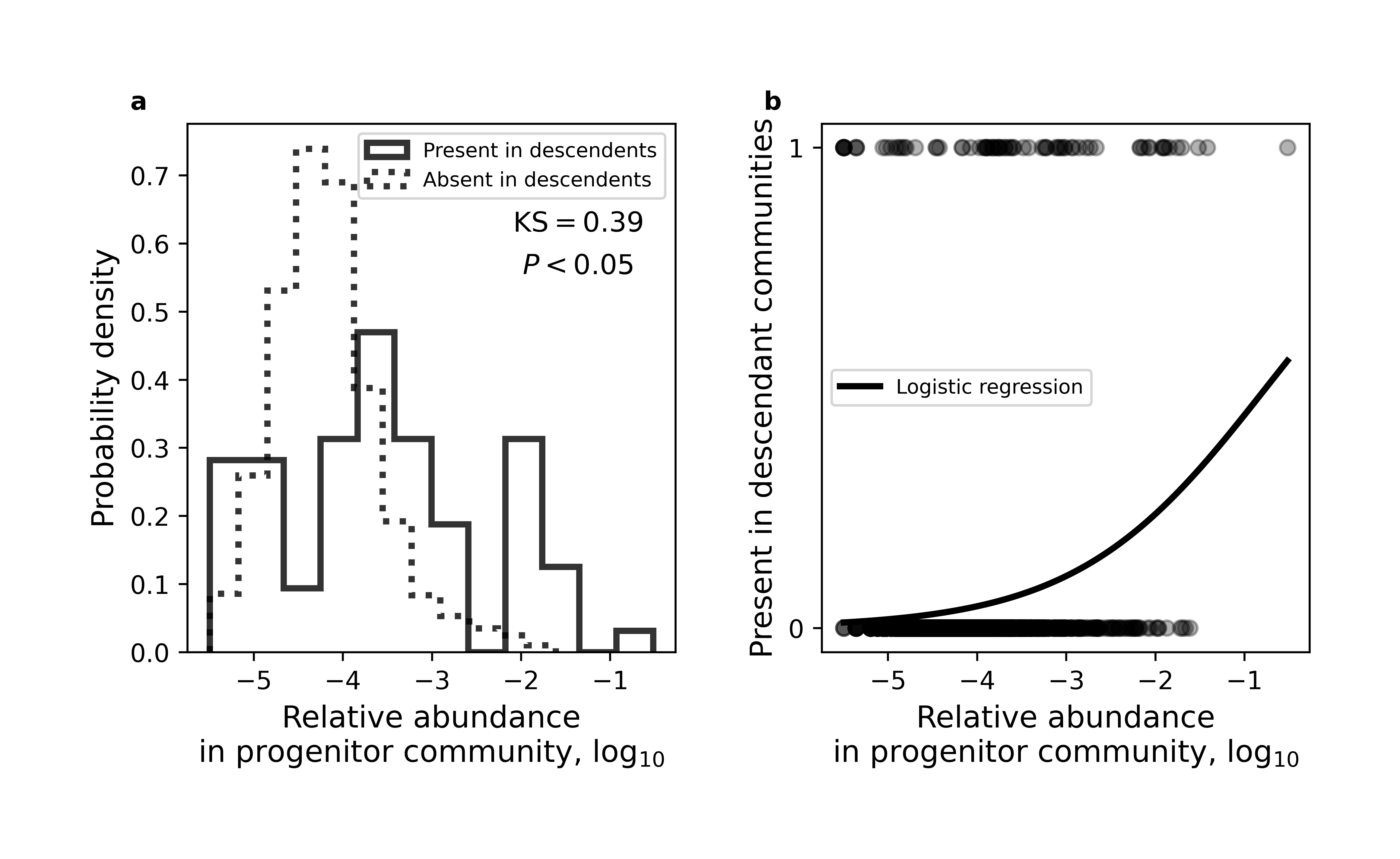

Supplement: S6 Fig — a) An ASV is more likely to be present in the descendant communities if it has a higher relative abundance in the progenitor. b) This result implies that probability that an ASV has a non-zero carrying capacity is a function of its progenitor abundance, a relationship that can be modeled as a logistic regression. (TIFF) [file pcbi.1013044.s014.tiff]

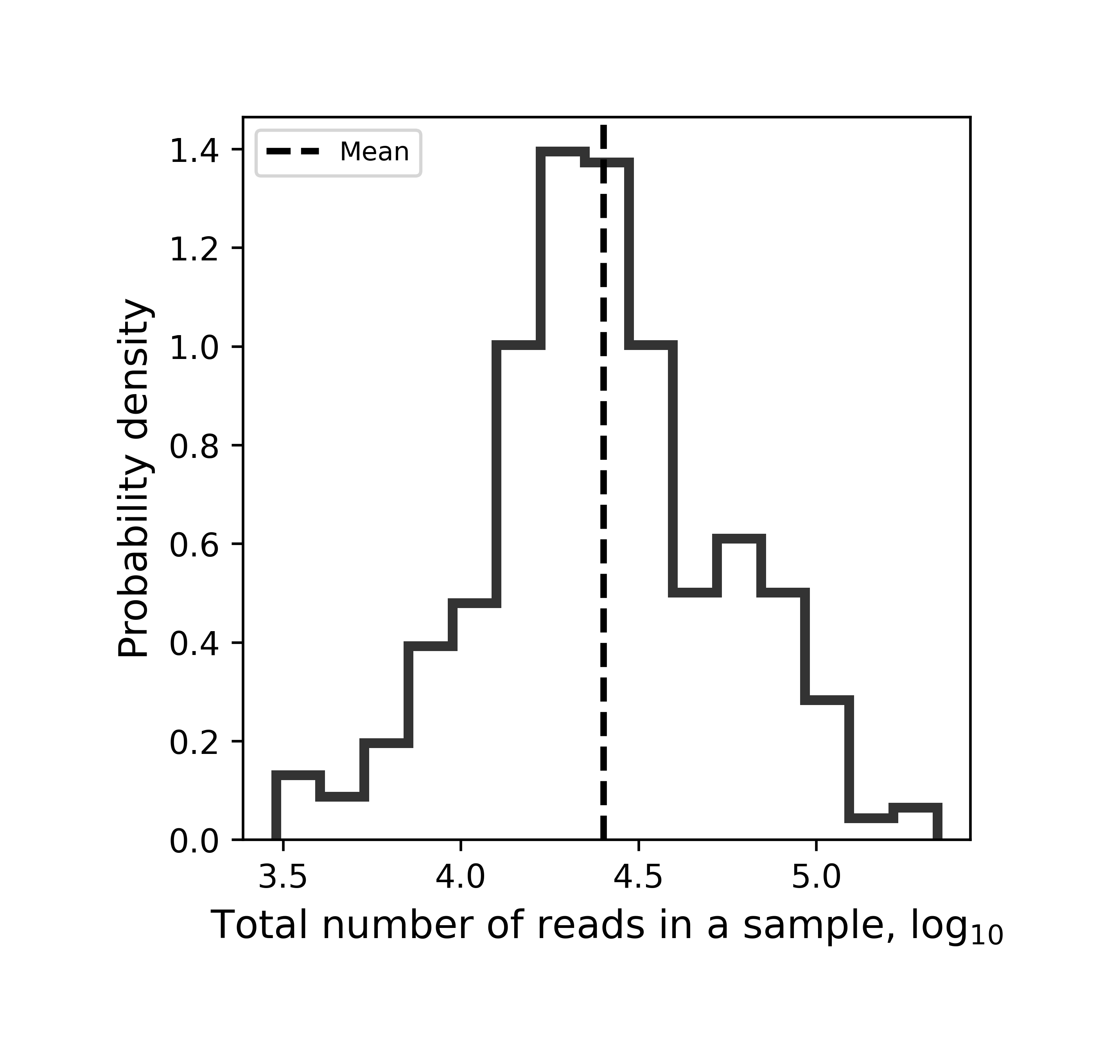

Supplement: S8 Fig — In our simulations the generation of reads from relative abundances was done as a multinomial sampling process, where the total number of reads of a given replicate at a given time point was drawn from the empirical distribution of total read counts. (TIFF) [file pcbi.1013044.s016.tiff]

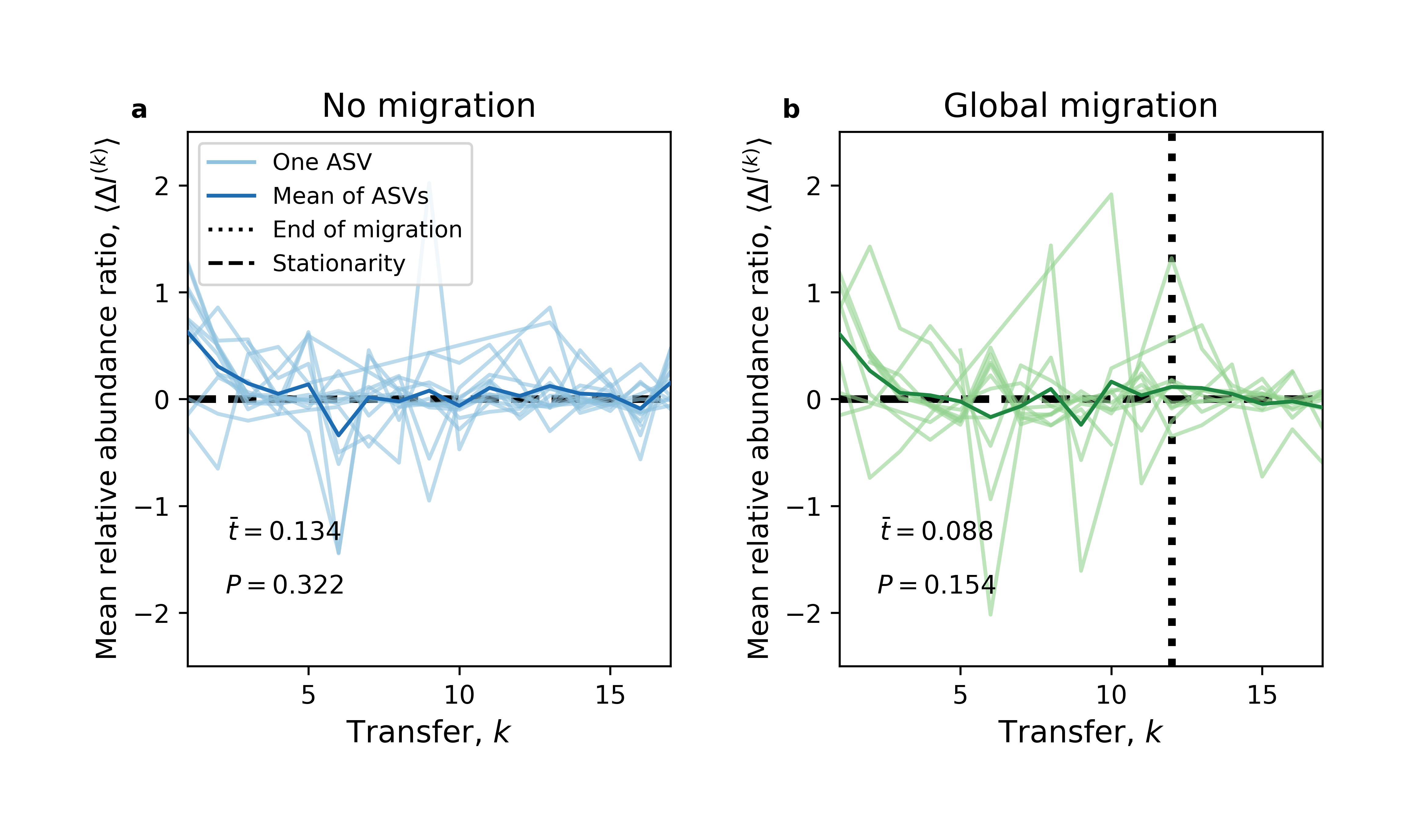

Supplement: S15 Fig — For both a) no and b) global migration the mean of Δℓ is initially higher than the stationary value of zero, though the mean relaxes to zero by transfer six for both treatments and does not appear to change after the cessation of global migration. This result is consistent with predicted consequences of global migration. (TIFF) [file pcbi.1013044.s023.tiff]
