# Supplementary material for: Macroecological patterns in experimental microbial communities
Source: PLoS Comput Biol. 2025 May 8;21(5):e1013044. doi: 10.1371/journal.pcbi.1013044 (PMC12112161; doi:10.1371/journal.pcbi.1013044)
Supplement: S1 Table — (PDF) [file pcbi.1013044.s024.pdf]

Macroecological patterns in experimental microbial communities: S1 Table

William R. Shoemaker[1,*], Álvaro Sánchez[2], and Jacopo Grilli[1]

1 Quantitative Life Sciences, The Abdus Salam International Centre for Theoretical Physics (ICTP), Trieste, 34151, Italy.
2 Instituto de Biología Funcional y Genómica, IBFG-CSIC, Universidad de Salamanca, 37007, Salamanca, Spain.

*** Contact:** `williamrshoemaker@gmail.com`

Migration treatment	Inoculation	Transfer(s)	# sequenced communities
No migration	Low	12	20
		18	92
		1-11, 13-17	20
No migration	High	12	4
		18	93
Regional	Low	12	92
		18	92
		1-11, 13-17	8
Global	Low	12	93
		18	93
		1-11, 13-17	3

Table S1. The number of replicate communities sequenced for a given treatment at a given transfer, summarized from [1].

References

1. Sylvie Estrela, Jean C. C. Vila, Nanxi Lu, Djordje Bajić, Maria Rebolleda-Gómez, Chang-Yu Chang, Joshua E. Goldford, Alicia Sanchez-Gorostiaga, and Alvaro Sanchez. Functional attractors in microbial community assembly. *Cell Systems*, 13(1):29–42.e7, January 2022.
