# Supplementary material for: Macroecological patterns in experimental microbial communities
Source: PLoS Comput Biol. 2025 May 8;21(5):e1013044. doi: 10.1371/journal.pcbi.1013044 (PMC12112161; doi:10.1371/journal.pcbi.1013044)
Supplement: S2 Table — The percent of communities belonging to a given attractor for each migration treatment. (PDF) [file pcbi.1013044.s025.pdf]

Macroecological patterns in experimental microbial communities: S2 Table

William R. Shoemaker[1,*], Álvaro Sánchez[2], and Jacopo Grilli[1]

1 Quantitative Life Sciences, The Abdus Salam International Centre for Theoretical Physics (ICTP), Trieste, 34151, Italy.
2 Instituto de Biología Funcional y Genómica, IBFG-CSIC, Universidad de Salamanca, 37007, Salamanca, Spain.

*** Contact:** `williamrshoemaker@gmail.com`

		% communities in a given attractor	
Transfer regime	Inoculation	Alcaligenaceae	Pseudomonadaceae
No migration	Low	70.7	29.3
No migration	High	0.0	100.0
Regional	Low	4.4	95.6
Global	Low	100.0	0.0

Table S1. The percent of communities belonging to a given attractor for each migration treatment, as described in [1].

References

1. Sylvie Estrela, Jean C. C. Vila, Nanxi Lu, Djordje Bajić, Maria Rebolleda-Gómez, Chang-Yu Chang, Joshua E. Goldford, Alicia Sanchez-Gorostiaga, and Alvaro Sanchez. Functional attractors in microbial community assembly. *Cell Systems*, 13(1):29–42.e7, January 2022.
