# Supplementary material for: Macroecological patterns in experimental microbial communities
Source: PLoS Comput Biol. 2025 May 8;21(5):e1013044. doi: 10.1371/journal.pcbi.1013044 (PMC12112161; doi:10.1371/journal.pcbi.1013044)
Supplement: Attachment — Submitted filename: point_by_point.pdf [file pcbi.1013044.s028.pdf]

Reviewer comments
Reviewer #1

The manuscript investigates whether broader patterns observed in the ecology hold when examining experiments in the laboratory. As the authors mention, this is an important step towards enabling relevant, causal studies using experimental ecology. For this, the manuscript studies patterns emerging from the stochastic logistic model (SLM) and uses high-replicate assembled communities of soil bacteria in a controlled experimental setting.
The manuscript is thorough and well-written, and it follows a logical progression. Necessary information regarding the motivations, the hypotheses, the model assumptions, and the experimental details are included, further strengthening the manuscript. Especially, I appreciate that the authors have included the detailed derivations related to their patterns of interest in the Methods section. These derivations serve as an important point of reference, without disrupting the flow of the main text.

In my opinion, this manuscript is an important contribution to the field and will be a very beneficial stepping-stone to enable future work in this direction. I only have a few minor suggestions, listed below.

- **Author response:** We thank the Reviewer for their positive comments.

Minor comments
1. For Fig 2c, the lower trend of the predicted pattern is not captured in the experimental data. I think it would be helpful to discuss why that might be.

- **Author response:** The author raises an important point. The left side of Fig. 2c represents a cutoff where mean abundances cannot be observed due to finite sampling. This cutoff was originally identified in the natural communities examined in Grilli (2020), motivating the development of a sampling-aware form of the lognormal Mean Abundance Distribution (Eq. 8 in the revised manuscript). We clarified this point in the text, as we now explicitly state the existence of this cutoff in the Methods (line 209-212) and the legend of Fig. 2.

2. In Fig 2d, please include more description of the two free parameters. In its current form, it takes too much extra effort for the reader to find the relevant information (which is needed to interpret Fig 2d).

- **Author response:** We have revised the legend of Fig. 2 to clarify the meaning of the two parameters in Fig. 2d and the Results (lines 395-398).

3. In Fig 4 caption, I recommend mentioning what the optimal parameters represent. The values are mentioned in the figure and the description is included in the text, but I think it would be helpful to just briefly mention that they are from fitting the model into data.

- **Author response:** We have added additional information on the parameters to the caption of Fig. 4. We have also added this information to the captions of all main manuscript figures that used ABC-selected parameters (Figs. 5-7)

4. In Figures 4, 5, and 6, I suggest using a consistent range for the x axis (and the y axis) across different cases (e.g. no, regional, and global migration) to make comparisons easier.

- **Author response:** In the revised manuscript we now use the same range for the x-axis of the AFDs in Fig. 4a-c, for both axes of Taylor's Law in Fig. 5a-f, and both axes of the mean relative abundance and mean relative abundance ratio in Fig. 6a,b,d,e.

Reviewer #2
In this manuscript, the authors present what appears to be the first analysis in the literature of macroecological laws applied to experimental microbial communities grown in laboratory conditions. These communities, isolated from soil, were cultivated in microcosms containing a single carbon source over a 48-hour period, with the experimental results previously published in an earlier study.

The manuscript focuses on two main objectives. The first is to verify the validity (or potential variation) of three macroecological laws recently proposed by the last author of this manuscript for microbial communities from diverse origins. Specifically, these laws include: (1) the abundance-fluctuation distribution of bacterial species follows a gamma distribution, (2) mean abundances and variances are not independent but are linked by Taylor's law, and (3) mean abundances are distributed according to a lognormal distribution.

The first conclusion is that these laws do qualitatively hold in in-lab grown communities with relatively good accuracy even if the curves are not as precise as in "wild" communities. On the other hand, migration (especially regional migration) can affect the shape of the abundance fluctuation distribution. The observation of this shift in AFD is attributed to the possibility of alternative states, where some species can have large abundances in some states and low in others, implying some sort of ergodicity breaking.

The first conclusion is that these laws qualitatively hold in laboratory communities with relatively good accuracy, although the curves are not as precise as those observed in "wild" communities. However, migration, particularly regional migration, can influence the shape of the abundance-fluctuation distribution (AFD). This shift in AFD is attributed to the possibility of alternative states, where certain species exhibit high abundances in some states and low abundances in others, suggesting a form of ergodicity breaking.

The second focus is to determine whether the stochastic logistic model (SLM), which, along with additional assumptions, has been used to justify the aforementioned macroecological laws, can be adapted to model experimental setups that incorporate two types of migration. In the first type, termed regional migration, new individuals from a source (mother) community are added

after a serial dilution, assuming a typical mainland-island dynamic. In the second type, referred to as global migration, multiple communities evolve in parallel and exchange individuals during serial dilution steps. The main conclusion is that the SLM is able to qualitatively explain the observed patterns in both cases. However, some subtle differences arise in quantitative comparisons, particularly in the case of regional migration.
Overall, the paper is well-written, with careful statistical and mathematical analyses. The results are robust, and the conclusions are both relevant and meaningful. Therefore, I fully support the publication of this manuscript in PCB.

- **Author response:** We thank the Reviewer for their thoughtful summary of the manuscript, their positive remarks, and the useful suggestions.

However, I encourage the authors to consider the following suggestions for potential improvements or clarifications.
1. Could the authors elaborate on why it was necessary to redo the analyses of Estrela et al. using ASVs? Including a brief comment or discussion on the distinction between ASVs and OTUs would provide helpful context.

- **Author response:** Estrela et al. used ASVs. However, they did not obtain ASV read counts that were equal to one. This is because default DADA2 settings do not attempt to infer read counts of one (n=1 in our manuscript) due to the existence of sequencing error. This truncation limits the range of read counts that can be inferred from empirical data, effectively throwing away data. However, DADA2 can be used to accurately infer read counts of one if it is provided with greater computational resources. Therefore, we re-ran DADA2 using the package's procedure for inferring read counts of one by pooling observations across samples to get a reliable estimate of the rate of sequencing error. By inferring read counts of one we were able to analyze the full distribution of read counts for each ASV. In the revised manuscript we provide this explanation along with a brief comment on the distinction between ASVs and OTUs with appropriate references (lines 119-126).

2. The fit shown in Fig. 2, particularly the one to the gamma distribution, doesn't appear to be very good. Have the authors tested any alternative fits that might provide a better correction to the gamma distribution? Similarly, for the third law, while it seems marginally compatible with a lognormal distribution, it could also potentially follow a power law. Please comment on this.

- **Author response:** We thank the reviewer for their comments. While we were performing additional analysis in the data used for Fig. 2a, we realized that there was a discrepancy in the code used to generate Fig. 2a, as the AFDs of each ASV were not rescaled appropriately. The log relative abundance of a given ASV should have been rescaled by the mean and standard deviation of that ASV, as previously described in the text (now on lines 342-343). Upon correction, we found that AFDs across treatments exhibited a better data collapse, meaning that a gamma distribution was able to capture this collapse better

than previously shown. The responsible code was isolated to Fig. 2a and affected no other analysis.

It is however still true that the gamma distribution does not capture the right tail of the empirical distribution. We believe that the discrepancy shown Fig. 2a is due to the existence of alternative stable states in the no migration treatment. However, given the improved data collapse and that we were able to predict the occupancy of separate ASVs using the form of the gamma distribution that explicitly accounts for sampling effort with reasonable accuracy (Fig. 2b; S1a), we believe that the gamma provides a reliable description. As a point of comparison, prior investigations on natural communities found that a gamma distribution was able to predict occupancy while a lognormal distribution consistently failed to predict both the AFD and occupancy (Supplementary Figs. 3, 4 in Grilli, 2020). We include these points in the revised manuscript (lines 345-349; 374-377).

Regarding the MAD in Fig. 2c, lognormal distributions and power law distributions can appear similar when the lognormal scale parameter s is large. Given that the fitted lognormal does not appear linear on a log-log scale and that the scale parameter s continues to decrease with time across experimental conditions (Fig. 2d), we believe that the lognormal serves as a reliable descriptor of the empirical MAD.

The fitted lognormal distribution may not appear to capture the data because we rescaled the empirical MAD of each treatment in order to present all treatments within a single figure. While we argue that the MADs of all treatments follow a lognormal, inferred parameter values indicate that not all treatments can be fully described by a lognormal with the same parameters (Fig. 2d). Therefore, in the revision we provide a figure of the MAD of the treatment with the most ASVs (i.e., regional migration) with a lognormal fit (Fig. S2). We see that the MAD is not linear on a log-log scale, suggesting that a power law would not provide an appropriate description of the data. We include these points in the revised manuscript (lines 401-407, Fig S2 legend).

3. In recent work (Proceedings of the National Academy of Sciences, 120 (44), e2215832120) it was argued that demographic noise is the best proxy for describing the dynamics and steady-state distribution of microbial communities. Could the authors elaborate a little bit on this point and specify why they favor environmental noise in their current analyses?

- **Author response:** The reviewer raised an important point regarding the form of multiplicative noise that best describes microbial ecological dynamics. We are aware of the conclusions in George and O'Dwyer (2023). While they provide a different interpretation, their mathematical model is fundamentally the same as the migration-birth-demographic noise model that has been examined elsewhere (Grilli, 2020; Brigatti and Azaele, 2025). It is however not obvious how to extend this model to our experimental setting, as the existence of a non-trivial steady-state abundance depends on there being a non-zero migration rate. Alternatively stated, if this model was valid, we would expect to see either unbounded growth or a steady-state mean abundance of zero for experimental treatments where migration was absent.

Multiple ecological models have been proposed as explanations for a variety of microbial macroecological patterns A systematic study of the empirical microbial patterns that can and cannot be explained by a given ecological model has not been done, but at present, we believe that the SLM has explained the largest number of empirical patterns, can serve as a valid model for natural communities, and can be directly applied to experimental communities.

We have summarized the above points in the Materials and Methods of the revised manuscript (lines 177-188).

4. I find the Materials and Methods section quite lengthy and, at times, tedious. While I agree that all relevant details should be included, would it be possible to condense this section? Alternatively, secondary details could be moved to the appendices or supplementary information (SI).

- **Author response:** We understand the reviewer's request. In the revised manuscript we sought to strike a balance between requests to elaborate on certain points but reduce the overall length of the Materials and Methods. Specifically, we moved the justification for our global migration predictions to the supplement, cutting ~1 page from the manuscript (now in S7 Text). We have also reduced the size of the "Regional migration statistics" subsection.

5. Could the authors make an additional effort to condense and distill their findings in the Result section, presenting them in a more straightforward manner? The reader needs to remain highly focused in order not to get lost in the variety of migration forms, experiment-agnostic versus experiment-specific patterns, and the various deviations (or lack thereof) from SLM predictions.

- **Author response:** We understand that there are sections of the Results that are difficult to read. We have worked through the Results to identify opportunities for clarification, moving certain points to the Supplemental Text while reducing the length of the text in the Results.

6. I do not find the discussion about alternative stable states particularly clear. While I have no objections to the idea, I'm convinced the authors could present a stronger case to better illustrate the existence of such alternative states. In particular, I don't fully understand how the SLM, even when studied under the serial dilution and migration setup, can lead to a bimodal distribution of species abundances. Could you please make it more transparent for the readers?

- **Author response:** The reviewer is correct in that the SLM presented in this study does not incorporate alternative stable-states. We now clarify this point in the Discussion. We have also rewritten the part of the Discussion that summarizes a prior research effort that incorporated alternative stable-states into the SLM, which was tested on natural microbial communities with highly resolved temporal sampling (i.e., a number of temporal samples much greater than the 18 in the experiment we examined; lines 622-628).

7. Similarly, do the authors provide a strong explanation for why the exponents of Taylor's law change depending on the type of experiment, as reported in Fig. 5? More broadly, can any meaningful interpretation be made of the slope value?

- The ecological meaning of the exponent of Taylor's law can be understood by 1) inferring the value of the exponent from empirical data and then 2) examining how that value relates to the mean and variance of a given ecological model (e.g., the stationary distribution of the SLM). For timeseries of natural microbial communities the value of the exponent is close to two. Under the stationary solution of the SLM (i.e., no time dependence), the meaning of an exponent of two is that the strength of environmental noise (σ) is constant across community members.

  In experimental communities we found that the exponent varied by migration treatment. However, the time-dependent solution of the SLM, while known, is unwieldy, making it difficult to use the time-dependent mean and variance to glean ecological insight into the exponent of Taylor's Law as well as incorporate additional experiment-specific details (S5 Text). Therefore, we elected to perform simulations to determine how the exponent changed before and after the cessation of migration manipulations across two SLM parameters: the strength of environmental noise (σ) and the timescale of growth (τ) (Fig. S9). We have clarified these points in the main manuscript (lines 197-202) and the supplement (lines 122-128).

8. When the authors state, "Our results demonstrate that the AFD and Taylor's Law were primarily informative of the effects of regional (island-mainland) migration," do they imply that by empirically determining these patterns in real communities, one can draw conclusions about the type of migration dominating the community? Please clarify.

- **Author response:** The reviewer raises an interesting point regarding the applicability of conclusions drawn from laboratory community assembly experiments to natural communities. It is possible that natural communities that experience high rates of island-mainland migration will exhibit the changes in the AFD and Taylor's Law that we observed in experiments. However, a key parameter that we included in our simulations was the number of migrants transferred from the progenitor community to assembled communities. We knew the value of the parameter because it was chosen and implemented by the experimenters. In natural systems that parameter is not always known, though it could be estimated by tracking the rate that cells enter and exit the system. If such measurements were obtained for a variety of natural systems, one could investigate the dependency of macroecological patterns on the migration rate. We summarize these points in the Discussion of the revised manuscript (lines 675-683).

9. The meaning of the vertical lines in Fig.4 is not specified in the figure caption

- **Author response:** In the revised manuscript we describe the dashed and solid vertical lines in the caption of Fig. 4.

In summary, I greatly appreciate the research and the results, but I believe the authors could improve the presentation of their findings and structure the manuscript in a more straightforward manner.

Reviewer #3

In this study, the authors bridge macroecological patterns with experimental ecology by applying specific ecological theory models. The manuscript presents a compelling premise: that a model such as SML, when applied to microbial community experiments in microcosms, can reveal ecological patterns similar to those observed in nature and therefore bridge macroecology with experimental ecology.

While the article is generally well written, a gap emerges in the description of the previously published experiment from which all data were used. The experiment is not fully explained, requiring the reader to consult another article for essential details. Instead, key information—such as the general premise of the experiment, its design rationale, and the core concepts it was intended to test—should be incorporated into the introduction. Providing this context would give readers a clearer understanding of why this particular experiment is well-suited as a bridging study between macroecological patterns and experimental ecology.

- **Author response:** We thank the Reviewer for their comments. In our original submission we sought to provide sufficient information about the experiment through a brief summary of the experiment in the main manuscript, two conceptual diagrams illustrating core elements of the experimental design, and a more detailed, technical summary of the experiment in the Supplemental Text. In the revised manuscript we have added to these efforts by describing the motivation and design of the experiment in the final paragraph of the Introduction.

Minor Comments:
1. Line 85 – You briefly describe the experiment in citation #1 and mention that all microcosms contained a single supplied carbon source. You then specify glucose (i.e., glucose), and Figure 1a also shows only glucose examples. Were all microcosms using glucose, or were different carbon sources included? Please clarify.

- **Author response:** All microcosms were grown on glucose. In the revised manuscript this detail is mentioned in the Introduction, Methods, Discussion, Fig. 1 caption of the main manuscript (lines 66, 93, 582) and the Supplemental Text (line 6).

2. Line 87 – Can you specify the dilution ratio of the aliquot? Was it a 1:10 dilution, 1:100, or another ratio?

- **Author response:** The dilution rate of the serial dilution transfer was 1:125 (D = 0.008). The dilution rate of allocating the migration pools among replicate communities was

504:60,000 ($D \approx 0.0084$). The dilution rates and how they were calculated are in Table 1 and described in the Supplemental Text. In the revised manuscript we now explicitly state the dilutions and cite the table when the dilutions are first introduced in the Materials and Methods (lines 95, 108).

3. Line 107 – Did you sequence only the final transfer cycle of each microcosm? Please clarify.

- **Author response:** Not all microcosms were sequenced at every transfer. All microcosms were sequenced at transfers 12 and 18. A subset of those microcosms were sequenced at every transfer cycle. The number of microcosms sequenced for a given transfer cycle for a given treatment was provided in Table S1 and was cited in the Materials and Methods in the original manuscript. In the revised manuscript we now briefly summarize Table S1 in the Materials and Methods (lines 123-126).

4. Line 557 – What exactly do you mean by "large ecological variation"? Could you be more specific?

- **Author response:** In the revised manuscript we now specify that ecological variation refers to variation in abundance over several orders of magnitude (line 582).

References

Brigatti, E., and S. Azaele. "Growth-rate distributions of gut microbiota time series." Scientific Reports 15.1 (2025): 2789.

George, Ashish B., and James O'Dwyer. "Universal abundance fluctuations across microbial communities, tropical forests, and urban populations." Proceedings of the National Academy of Sciences 120.44 (2023): e2215832120.

Grilli, Jacopo. "Macroecological laws describe variation and diversity in microbial communities." Nature communications 11.1 (2020): 4743.
