# Supplementary material for: Macroecological patterns in experimental microbial communities
Source: PLoS Comput Biol. 2025 May 8;21(5):e1013044. doi: 10.1371/journal.pcbi.1013044 (PMC12112161; doi:10.1371/journal.pcbi.1013044)
Supplement: Attachment — Submitted filename: Report- Shoemaker 20 Feb.docx [file pcbi.1013044.s026.docx]

In this manuscript, the authors present what appears to be the first analysis in the literature of macroecological laws applied to experimental microbial communities grown in laboratory conditions. These communities, isolated from soil, were cultivated in microcosms containing a single carbon source over a 48-hour period, with the experimental results previously published in an earlier study.

The manuscript focuses on two main objectives. The first is to verify the validity (or potential variation) of three macroecological laws recently proposed by the last author of this manuscript for microbial communities from diverse origins. Specifically, these laws include: (1) the abundance-fluctuation distribution of bacterial species follows a gamma distribution, (2) mean abundances and variances are not independent but are linked by Taylor's law, and (3) mean abundances are distributed according to a lognormal distribution.

The first conclusion is that these laws do qualitatively hold in in-lab grown communities with relatively good accuracy even if the curves are not as precise as in "wild" communities. On the other hand, migration (especially regional migration) can affect the shape of the abundance fluctuation distribution. The observation of this shift in AFD is attributed to the possibility of alternative states, where some species can have large abundances in some states and low in others, implying some sort of ergodicity breaking.

The first conclusion is that these laws qualitatively hold in laboratory communities with relatively good accuracy, although the curves are not as precise as those observed in "wild" communities. However, migration, particularly regional migration, can influence the shape of the abundance-fluctuation distribution (AFD). This shift in AFD is attributed to the possibility of alternative states, where certain species exhibit high abundances in some states and low abundances in others, suggesting a form of ergodicity breaking.

The second focus is to determine whether the stochastic logistic model (SLM), which, along with additional assumptions, has been used to justify the aforementioned macroecological laws, can be adapted to model experimental setups that incorporate two types of migration. In the first type, termed regional migration, new individuals from a source (mother) community are added after a serial dilution, assuming a typical mainland-island dynamic. In the second type, referred to as global migration, multiple communities evolve in parallel and exchange individuals during serial dilution steps. The main conclusion is that the SLM is able to qualitatively explain the observed patterns in both cases. However, some subtle differences arise in quantitative comparisons, particularly in the case of regional migration.

Overall, the paper is well-written, with careful statistical and mathematical analyses. The results are robust, and the conclusions are both relevant and meaningful. Therefore, I fully support the publication of this manuscript in PCB.

However, I encourage the authors to consider the following suggestions for potential improvements or clarifications.

- Could the authors elaborate on why it was necessary to redo the analyses of Estrela et al. using ASVs? Including a brief comment or discussion on the distinction between ASVs and OTUs would provide helpful context.

- The fit shown in Fig. 2, particularly the one to the gamma distribution, doesn't appear to be very good. Have the authors tested any alternative fits that might provide a better correction to the gamma distribution? Similarly, for the third law, while it seems marginally compatible with a lognormal distribution, it could also potentially follow a power law. Please comment on this.

- In recent work (Proceedings of the National Academy of Sciences, 120 (44), e2215832120) it was argued that demographic noise is the best proxy for describing the dynamics and steady-state distribution of microbial communities. Could the authors elaborate a little bit on this point and specify why they favor environmental noise in their current analyses?

- I find the Materials and Methods section quite lengthy and, at times, tedious. While I agree that all relevant details should be included, would it be possible to condense this section? Alternatively, secondary details could be moved to the appendices or supplementary information (SI).

- Could the authors make an additional effort to condense and distill their findings in the Result section, presenting them in a more straightforward manner? The reader needs to remain highly focused in order not to get lost in the variety of migration forms, experiment-agnostic versus experiment-specific patterns, and the various deviations (or lack thereof) from SLM predictions.

- I do not find the discussion about alternative stable states particularly clear. While I have no objections to the idea, I’m convinced the authors could present a stronger case to better illustrate the existence of such alternative states. In particular, I don't fully understand how the SLM, even when studied under the serial dilution and migration setup, can lead to a bimodal distribution of species abundances. Could you please make it more transparent for the readers?

- Similarly, do the authors provide a strong explanation for why the exponents of Taylor's law change depending on the type of experiment, as reported in Fig. 5? More broadly, can any meaningful interpretation be made of the slope value?

- When the authors state, "Our results demonstrate that the AFD and Taylor’s Law were primarily informative of the effects of regional (island-mainland) migration," do they imply that by empirically determining these patterns in real communities, one can draw conclusions about the type of migration dominating the community? Please clarify.

- The meaning of the vertical lines in Fig.4 is not specified in the figure caption

In summary, I greatly appreciate the research and the results, but I believe the authors could improve the presentation of their findings and structure the manuscript in a more straightforward manner.
